# Loss of thyroid hormone receptor interactor 13 inhibits cell proliferation and survival in human chronic lymphocytic leukemia

**DOI:** 10.18632/oncotarget.16038

**Published:** 2017-03-09

**Authors:** Keshu Zhou, Wentao Zhang, Qing Zhang, Ruirui Gui, Huifang Zhao, Xiaofei Chai, Yufu Li, Xudong Wei, Yongping Song

**Affiliations:** ^1^ Department of Hematology, Affiliated Cancer Hospital of Zhengzhou University, Henan Cancer Hospital, Zhengzhou, People's Republic of China; ^2^ Armed Police Forces Hospital of Henan, People's Republic of China

**Keywords:** TRIP13, chronic lymphocytic leukemia, c-MYC, PUMA, apoptosis

## Abstract

**Background:**

The genetic regulation of apoptosis and cell proliferation plays a role in the growth of chronic lymphocytic leukemia (CLL), the most common form of leukemia in the Western hemisphere. Although thyroid hormone receptor interactors (TRIPs) are known to play roles in cell cycle, the potential involvement of the novel family member TRIP13 in CLL has not yet been investigated.

**Methods:**

Quantitative PCR (qPCR) was used to detect expression of TRIP13 in 36 CLL patients and 33 healthy donors CD19+ B cells. Loss-of-function (siRNA) assays were used to alter TRIP13 expression levels. The effect of TRIP13 on cell proliferation and apoptosis was measured by MTT, Annexin V-based flow cytometry and Caspase 3/7 activity assay. Affymetrix GeneChip and Ingenuity Pathway Analysis (IPA) were used to describe an overview of TRIP13 potential biological function and downstream pathways. Dual-luciferase reporter assay was performed to assess the promoting effect of c-MYC on TRIP13 transcription.

**RESULTS:**

The qPCR data showed that TRIP13 is significantly over-expressed in CLL patients. Microarray analyses indicated that the biological function of TRIP13 in CLL is majorly cell apoptosis and cell proliferation associated. TRIP13 siRNA expressing cells exhibited a slower cell proliferation rate and underwent apoptosis compared with control cells. TRIP13 knockdown induced CLL cells apoptosis through PUMA independent of p53. TRIP13 up-regulation is induced by c-MYC dependent transcriptional activation.

**Conclusion:**

Overall, our data suggest the bio-function of TRIP13 in CLL cell for the first time, and that this gene might be a therapeutic target for CLL.

## INTRODUCTION

Chronic Lymphocytic Leukemia (CLL) is an incurable B-cell malignancy and the most common form of leukemia in the Western hemisphere [1]. Recently, new therapeutic approaches, including chemical compound, stem cell transplantation and monoclonal antibody-based therapies, have shown increased response rates [2–8]. However, minor effects on CLL overall survival achieved [9]. Therefore, new therapeutic targets are clearly needed for CLL patients.

Thyroid hormone Receptor Interactor 13 (TRIP13 or HPV16E1BP) is the mouse orthologue of pachytene checkpoint 2 (Pch2), a protein-remodeling AAA+ ATPase and cell cycle checkpoint in mouse, yeast and Caenorhabditis elegans [10–13]. Most recently, relationship between TRIP13 and human cancer attract scientist's attention. In squamous cell carcinoma of the head and neck (SCCHN), TRIP13 over-expression leads to aggressive, treatment-resistant tumors and enhanced repair of DNA damage [14]. TRIP13 expression in combination with preoperative PSA level was able to predict recurrence of prostate cancer [15]. TRIP13 genomic copy number also changes in non-small cell lung cancer [16]. However, the biological function of TRIP13 in non-solid cancer especially in CLL as well as the underlying molecular mechanism remains largely unclear.

On the basis of TRIP13 mRNA level in 36 CLL patients and 33 healthy donors, we nominated TRIP13 may be an oncogene. In this study, we investigated the role of TRIP13 in CLL. Knockdown of TRIP13 in CLL cell lines leads to cell apoptosis and growth failure. Microarray analyses showed a change in the expression of signaling related genes in these cells, suggesting that the cell proliferation defects in TRIP13 knockdown CLL cells might be linked to a change in multiple pathways. On this basis, we further found that TRIP13 inhibition results in a PUMA induced cell apoptosis independent of p53. We also found that the proto-oncogene C-MYC can promote TRIP13 expression by regulating its transcription in CLL cells. These findings define a mechanism of pro-proliferative effect of TRIP13 in CLL and emphasize the importance of potential usage of TRIP13 for CLL therapeutic applications.

## RESULTS

### TRIP13 is up-regulated in chronic lymphocytic leukemia patients

**Figure 1:**
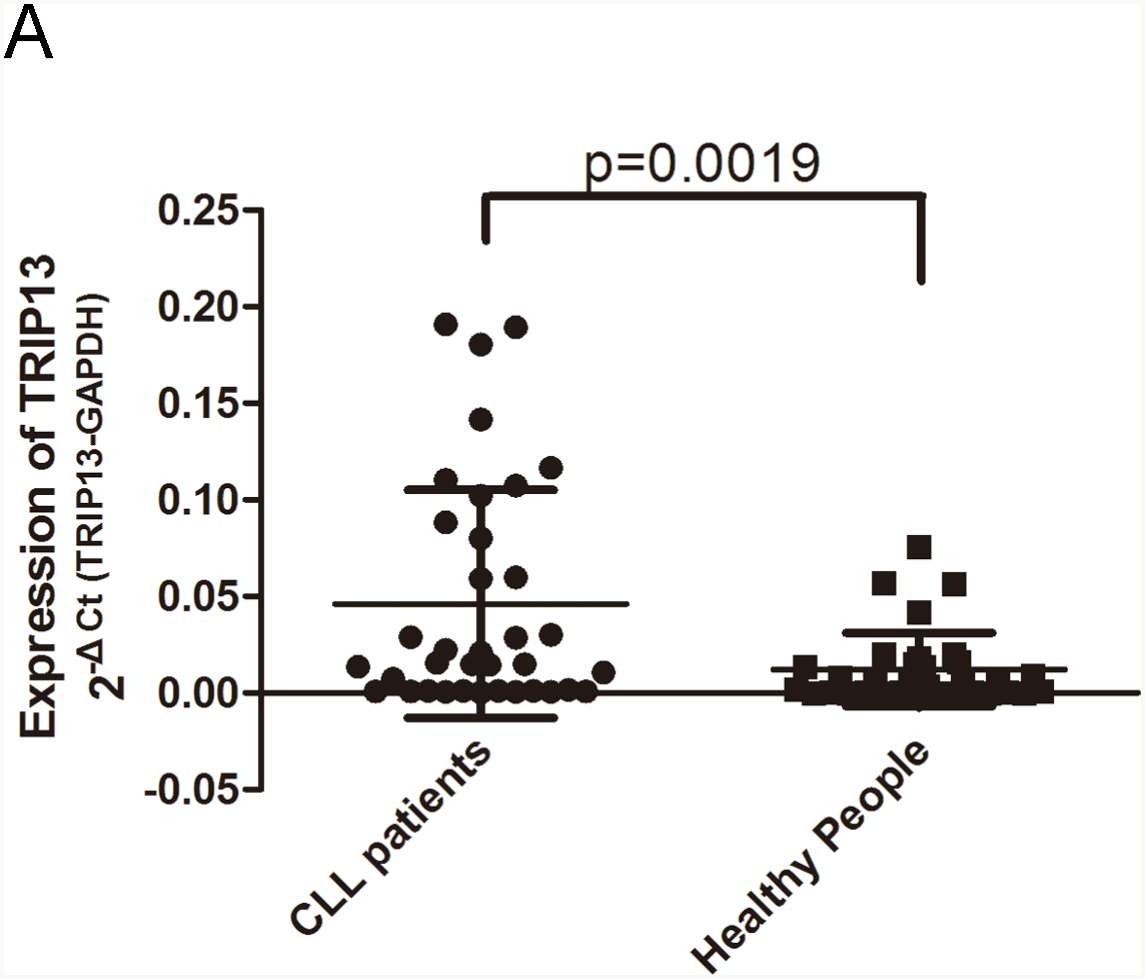
TRIP13 is up-regulated in chronic lymphocytic leukemia patients Expression of mature TRIP13 in human CLL cohort and healthy donors were determined by qPCR and normalized against an endogenous control GAPDH. **(A)** TRIP13 expression in 33 CLL patients and 36 healthy donors. TRIP13 expression levels were calculated by the TRIP13/GAPDH expression ratio (ie, 2^−ΔCt^). The mean TRIP13 expression levels in CLL patients and healthy donors were 0.048 and 0.012, respectively. Error bars indicate means ± SD. TRIP13 expression was significantly up-regulated in CLL patients (p = 0.0019, Mann–Whitney test).

We examined TRIP13 mRNA expression in CD19+ B cells isolated from 36 CLL patients and 33 healthy donors by qPCR. The average expression level of TRIP13 in CLL CD19+ B cells was 0.048 (2^−ΔCt (TRIP13-GAPDH)^) while the TRIP13 level in healthy CD19+ B cells was 0.012 (2^−ΔCt (TRIP13-GAPDH)^) (Figure 1A). The TRIP13 mRNA level was 4 fold higher in CLL patient CD19+ B cells than in the healthy person CD19+ B cells (p = 0.0019). Moreover, The Cancer Genome Atlas (TCGA) data showed that TRIP13 is frequently mutated or amplified in several kinds of cancer such as lung cancer and esophagus cancer but not in CLL (Supplementary Figure 1). The result indicated that TRIP13 dysregulation is most likely in transcriptional level but not in genomic level and may play a role in promoting CLL.

There are few CLL immortalized cell line models. Considering that Granta-519 and JVM-2 cells were more friendly to lentivirus and these two cell lines can be especially recommended for *in vivo* study of p53-mutated and p53-wild chronic lymphocytic leukemia and in which TRIP13 expression level are similar, these 2 cell lines were employed in the further study [17, 18].

### Knockdown of TRIP13 inhibited CLL cells growth *in vitro*

**Figure 2:**
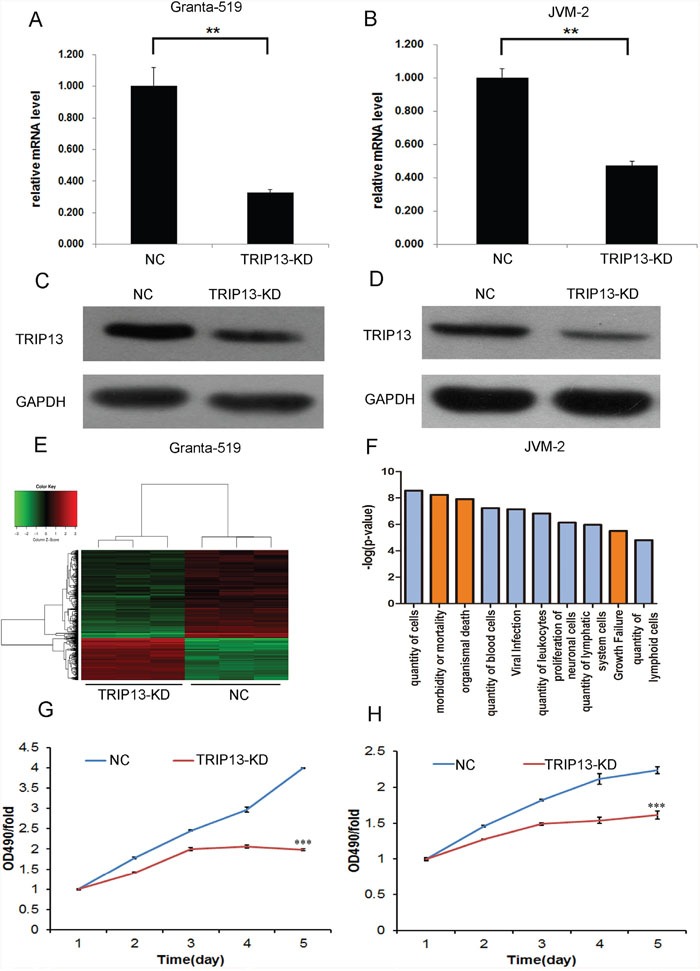
Knockdown of TRIP13 inhibited CLL cells growth *in vitro* Both of mRNA and protein level of TRIP13 knockdown were detected respectively by qPCR and western blot in Granta-519 and JVM-2 cells based on lentiviral-based shRNA. **(A)** and **(B)** Granta-519 and JVM-2 cells were infected with lentivirus expressing either TRIP13-siRNA or NC and cultured for 4 days. Then total RNA was extracted for reverse transcription and qPCR to quantify TRIP13 expression at mRNA level. TRIP13 mRNA level was normalized to GAPDH. Data shown are the mean ± S.D. of three independent experiments (**p<0.01, ttest). **(C)** and **(D)** TRIP13 expression at protein level was analyzed by western blot in Granta-519 and JVM-2 cells treated as described after 4 days of cell culture as well, total proteins were isolated, and TRIP13 protein was detected by specific TRIP13 antibody. GAPDH was used as an internal control. Widespread changes of gene expressions in JVM-2 cells with TRIP13 knockdown by microarray. **(E)** Heatmap representation of genes significant differential expressions in JVM-2 cells infected with lentivirus expressing either NC-siRNA or TRIP13-siRNA under the criteria p<0.05 and | fold change | >1.5. Genes and samples were listed in rows and columns, respectively. A color scale for the normalized expression data was shown at the bottom of the microarray heatmap (green represents downregulated genes while red represents upregulated genes). **(F)** Activated (red) and Inhibited (blue) TRIP13 related disease and function enrichment was analyzed based on IPA databases. Here, the ten significant-enriched diseases or functions based on a P value were shown. The statistical significance shown in X axis is represented by the inverse log of the P value. **(G)** and **(H)** The proliferation curve of TRIP13 siRNA infected Granta-519 and JVM-2 cells compared with control group, was determined by MTT assay (***p<0.001, ttest).

According to the above result, we decided to explore TRIP13 biological function through RNA interference. We did Lentivirus-mediated knockdown of TRIP13 in Granta-519 and JVM-2 cells. The lentivirus infection efficiency is above 85% for both TRIP13-KD lentivirus and Negative Control (NC) lentivirus, so that we can ensure the synchronization of all the following experiments (Supplementary Figure 2A and 2B). TRIP13 mRNA levels were assessed by quantitative qPCR. The results showed TRIP13-KD lentivirus infected cultures exhibited significantly reduced TRIP13 transcripts compared with cells infected with NC lentivirus (inhibitory efficiency in Granta-519 and JVM-2 is 67.3±1.9% and 52.8±2.6%) (p < 0.01, Figure 2A and 2B). The similar trend on TRIP13 protein levels was observed as on its mRNA levels by immunoblotting analysis in these two cell lines (Figure 2C and 2D).

Affymetrix GeneChip and Ingenuity Pathway Analysis (IPA) were then used to describe an overview of TRIP13 potential biological function. As shown in Figure 2E, 231 genes were up-regulated and 474 genes were down-regulated in TRIP13 knockdown JVM-2 cells compared with NC cells. IPA disease and function analysis demonstrated that TRIP13 is majorly in charge of cell quantity, cell death and growth especially in blood or lymphoid cells. As shown in Figure 2F, in the “quantity of cells”, “quantity of blood cells”, “quantity of leukocytes” functions were inhibited and “morbidity or mortality”, “organismal death” and “growth failure” functions were promoted in TRIP13 knockdown CLL cells. These results indicated that TRIP13 most likely play a role in promoting cell proliferation.

Granta-519 and JVM-2 cells infected with either TRIP13-KD lentivirus or NC lentivirus were seeded in 96-well plates, and cell growth was monitored by MTT every day for 5 days. Cell growth rate was defined as: cell count of Nth day/cell count of 1st day, where n = 2, 3, 4, 5. The results showed that down-regulation of TRIP13 decreased the total number of cells and cell growth rate was slowed down. The significance of 5^th^ day cell proliferative rate were p < 0.001 and p < 0.001 in Granta-519 and JVM-2 cells, respectively (Figure 2G and 2H). The BrdU incorporation DNA synthesis assay demonstrated that TRIP13 siRNA significantly reduced proliferation of JVM-2 (p < 0.01) and Granta-519 (p < 0.05) cells for 4 days (Supplementary Figure 3A and 3B).

### TRIP13 knockdown induced CLL cells apoptosis through PUMA independent of p53

**Figure 3:**
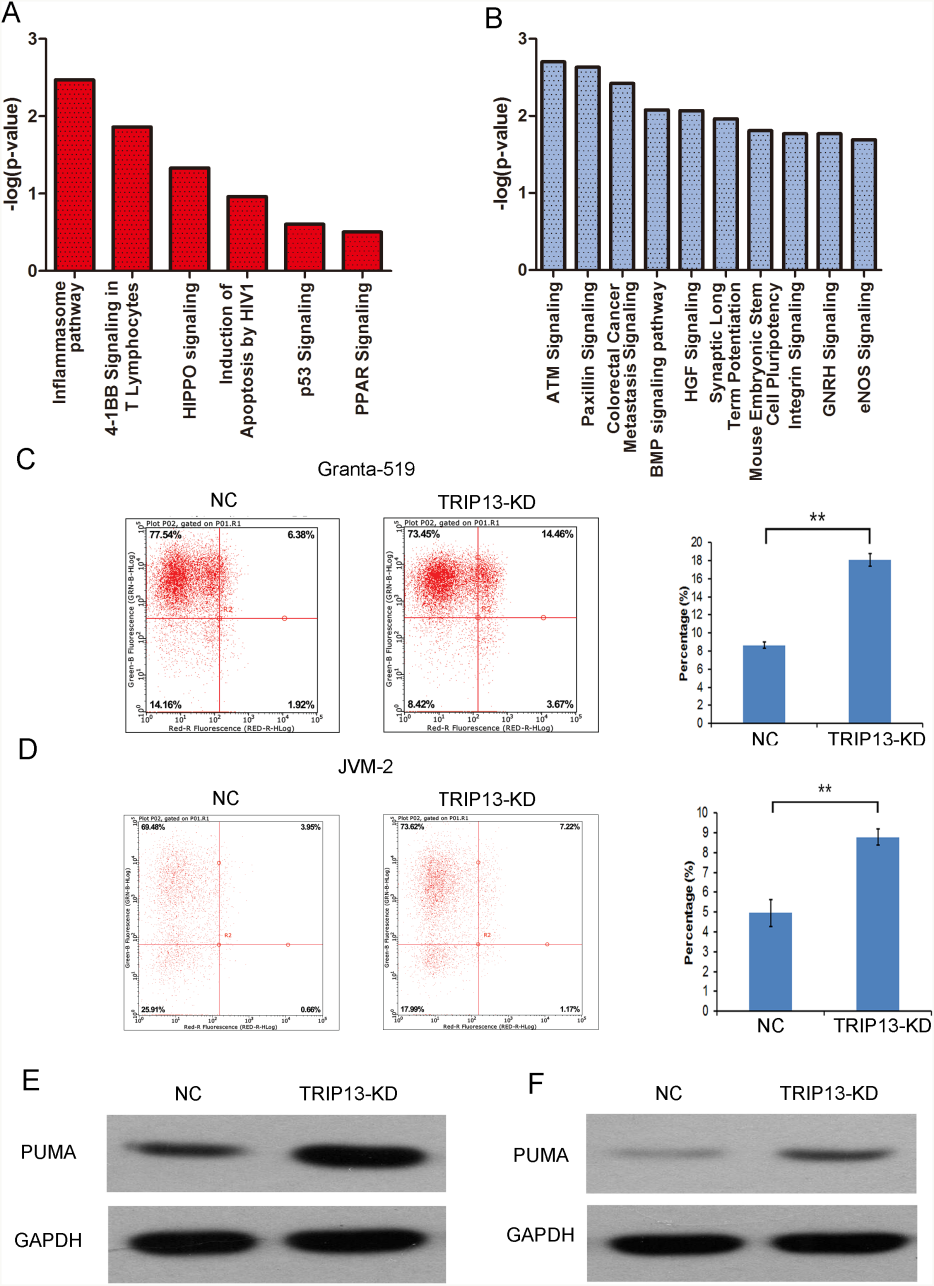
TRIP13 knockdown induced CLL cells apoptosis **(A)** and **(B)** Activated (red) and Inhibited (blue) TRIP13 related functional pathway enrichment was analyzed based on IPA databases. Here, the 6 significant activated and 10 significant inhibited pathway based on a P value were shown. The statistical significance shown in X axis is represented by the inverse log of the P value. **(C)** and **(D)** The apoptosis of Granta-519 and JVM-2 cells was induced by TRIP13-siRNA infection. Flow cytometry analysis of cell apoptosis reveals that the apoptosis rate of TRIP13 knockdown cells was significantly increased with respect to NC group (**p < 0.01, ttest). **(E)** and **(F)** The effects of TRIP13 knockdown on PUMA protein expression according to western blotting in Granta-519 and JVM-2 cells. GAPDH was used as a loading control.

The above results indicated that TRIP13 is critical for CLL cell proliferation. However, mechanisms underlying TRIP13-mediated CLL development are still unclear. To systematically explore the downstream pathways, the microarray data were analyzed by IPA “canonical pathway” module. The exported data showed that several critical pathways involved in cancer development and apoptosis such as “induction of apoptosis by HIV1”, “p53 signaling” and “PPAR signaling” were activated while pathways involved in DNA repairing and oncogenic function such as “ATM signaling” and “colorectal cancer Metastasis signaling” were inhibited by TRIP13 knockdown (Figure 3A and 3B).

Previous studies reported that TRIP13 promote cell oncogenic transformation through DNA repairing through cell pathways such as p31/MAD2 or ATM signaling [14, 19, 20]. We then focused on the mechanisms that TRIP13 played on CLL apoptosis. FACS analysis of Annexin V-stained cells demonstrated that the percentage of TRIP13-KD cells undergoing apoptosis (18.08±0.7% in Granta-519 and 8.78±0.42% in JVM-2) was significantly higher compared with the control group (8.64±0.34% in Granta-519 and 4.95±0.69% in JVM-2) (p < 0.01) (Figure 3C and 3D). Furthermore, we examined the activity of caspases 3 and 7 which play central roles in the execution-phase of cell apoptosis. Supplementary Figure 3C and 3D showed that TRIP13-KD resulted in a increasing caspase 3/7 activity in Granta-519 (198.43±3.04%, p < 0.001) and JVM-2 (205.6±0.85%, p < 0.001) cells. The western blot data showed that CHEK1 and Bcl-2 were decreased and Caspase 3 were increased in TRIP13-KD cells (Supplementary Figure 4). These data suggest that the effects of TRIP13 siRNA on the cell cycle checkpoint and apoptosis were likely dependent on the broader regulation of cellular functions.

**Figure 4:**
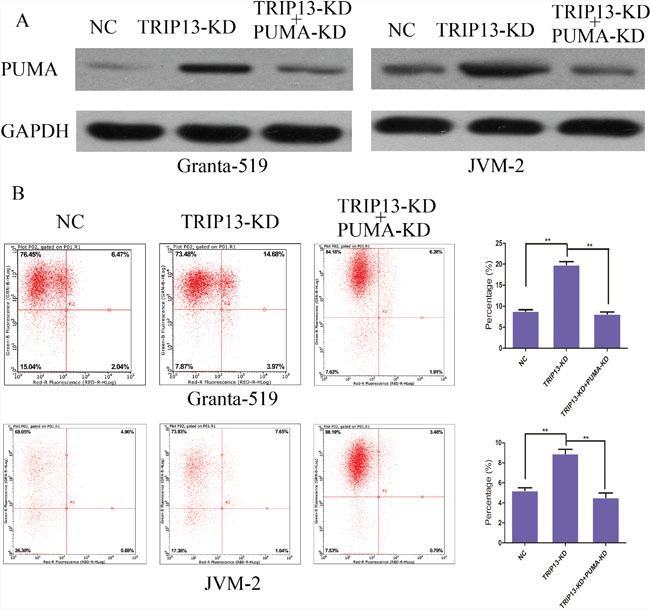
TRIP13 knockdown induced CLL cells apoptosis through PUMA independent of p53 **(A)** Protein level of negative control, TRIP13 knockdown and TRIP13 knockdown + PUMA knockdown groups were detected respectively by western blot in Granta-519 and JVM-2 cells. **(B)** The apoptosis of Granta-519 and JVM-2 induced by TRIP13-siRNA were rescued by PUMA knockdown. Flow cytometry analysis of cell apoptosis reveals that the apoptosis rate of TRIP13 knockdown + PUMA knockdown cells was significantly decreased with respect to TRIP13 knockdown group (**p < 0.01, ttest).

The above data indicated that TRIP13 was able to promote or keep CLL cell malignancy by inhibiting apoptosis. Interestingly, we found that PUMA protein levels were up-regulated in p53-mutated Granta-519 cells as well as in p53-wild JVM-2 cells respectively (Figure 3E and 3F). Hence, we hypothesized that TRIP13 knockdown induced CLL cells apoptosis through PUMA but independent of p53. We then silenced PUMA in TRIP13-KD cells. Figure 4B showed that knockdown PUMA reduced the apoptosis induction led by TRIP13-KD in Granta-519 and JVM-2 cells.

### C-MYC up-regulated TRIP13 transactivity

**Figure 5:**
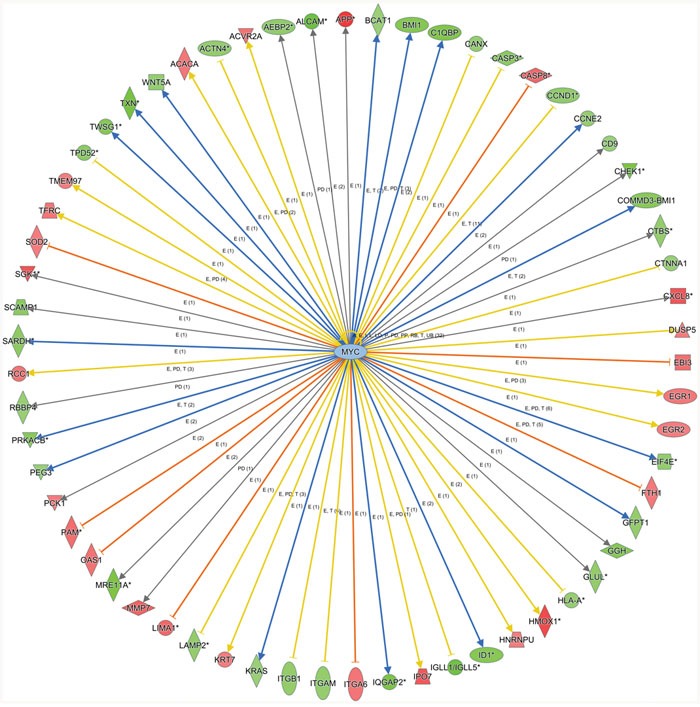
C-MYC is a potential upstream regulator of TRIP13 in CLL Knowledge-based interaction network of c-MYC and c-MYC targets after TRIP13 knockdown in JVM-2. The network was built based on the c-MYC interactome in the Ingenuity IPA database overlaid with microarray data from cells with a 1.5-fold change cut-off. The intensity of the node color indicates the degree of up- (red) or down- (green) regulation following AK4 overexpression in JVM-2 cells.

**Figure 6:**
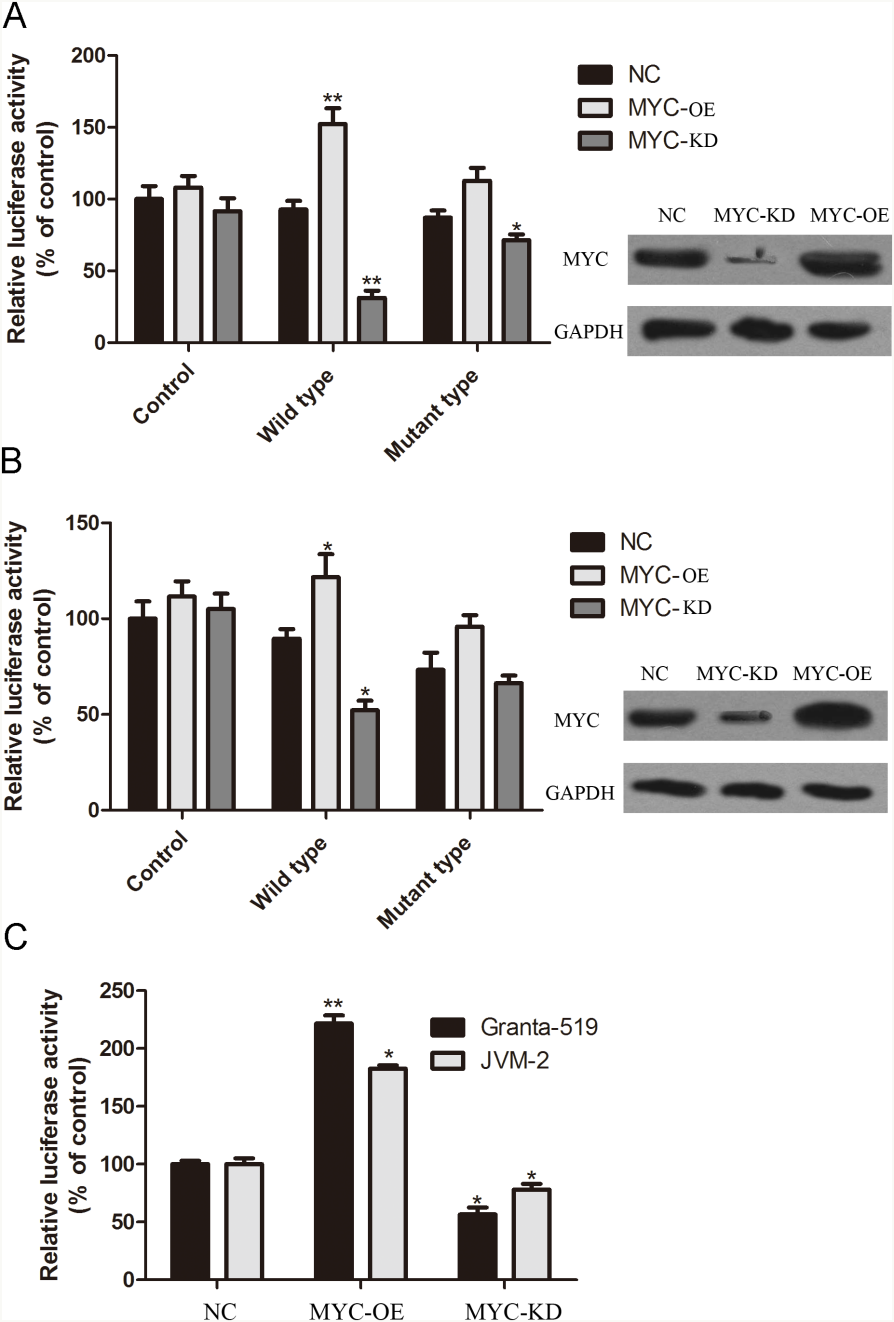
C-MYC up-regulated TRIP13 transactivity Dual-luciferase reporter assay was used to confirm the regulating effect of c-MYC on TRIP13. A 200bp containing these two binding sites sequences were inserted into PGL3-promoter plasmid before SV40 promoter. At the same time, a mutant type plasmid was constructed in which c-MYC binding sites were mutated following A to C and T to G rule. **(A)** and **(B)** wild type TRIP13 reporter activity was significantly increased in MYC-overexpressing cells and significantly decreased in MYC-silenced cells (**p < 0.01, *p < 0.05, ttest), whereas no significant changes were found in corresponding mutant type plasmid luciferase activity. **(C)** TRIP13 mRNA level were significantly increased in MYC-over-expression cells and decreased in MYC-silenced cells (**p < 0.01, *p < 0.05, ttest).

To shed light on the mechanism of TRIP13 up-regulation in CLL cells, IPA “Upstream analysis” module was performed to re-analyze the microarray data. According to the expression level of downstream target genes, the software predicted that c-MYC downstream signal pathway alone with its bio-function was inhibited after TRIP13 knockdown (Figure 5). C-MYC is a proto-oncogene that contributes to the genesis of CLL [21, 22]. Interestingly, we found that TRIP13 5′UTR region harbor 2 c-MYC binding sites (chr5: 884008-884028 strand (+) and chr5: 884117-884131 strand (-)) (Champion ChiP Transcription Factor Search Portal; http://www.sabiosciences.com/chipqpcrsearch.php?species_id=0&factor=c-Myc&gene=TRIP13&nfactor=n&ninfo=n&ngene=n&B2=Search) (Supplementary Figure 5). We then hypothesized that c-MYC is responsible for the over-expression of TRIP13 in CLL cells. The effect of c-MYC on the regulation of TRIP13 transactivity was examined. A 200bp containing these two binding sites sequences were inserted into PGL3-promoter plasmid before SV40 promoter. At the same time, a mutant type plasmid was constructed in which c-MYC binding sites were mutated following A to C and T to G rule. MYC was overexpressed and silenced in Granta-519 and JVM-2 cells by lentivirus mediated overexpression (OE) and silencing (KD). As shown in Figure 6A and 6B, wild type TRIP13 reporter activity was significantly increased in MYC-OE cells and significantly decreased in MYC-KD cells (increased to 152.3±11% and 121.7±12% in Granta-519 and JVM-2; decreased to 31.2±5% and 52.2±5% in Granta-519 and JVM-2), whereas no significant changes were found in corresponding mutant type plasmid luciferase activity in JVM-2 cells. Interestingly, there is significant alteration in mutant type luciferase activity in Granta-519. We hypothesized that their maybe exist other c-MYC related transcription regulator that exert regulatory effect on TRIP13 transcription. Importantly, TRIP13 mRNA level were significantly increased in MYC-over-expression cells (221.6±7% in Granta-519 and 182.6±3% in JVM-2) and decreased in MYC-silenced cells (56.6±6% in Granta-519 and 78±5% in JVM-2), further suggesting the specific effect of c-MYC on the TRIP13 transactivity (Figure 6C).

## DISCUSSION

The key findings of this study are that TRIP13 over-expression was frequently in human CLL and is significantly associated with progression of CLL. Decreased expression of TRIP13 induced apoptosis and inhibited the proliferation of CLL cells. We showed that c-MYC promotes TRIP13 expression by up-regulating its transcriptional activity. C-MYC/TRIP13/PUMA axis regulates CLL cell apoptosis independent of p53. This study provides new insights and strong evidences that TRIP13 over-expression plays important roles in promoting the tumorigenicity and progression of human CLL.

Although early studies reported that TRIP13 was a potential marker in lung cancer and prostate cancer, the direct link between TRIP13 and CLL has not been explored thoroughly. Recent studies also described the potential oncogenic roles of TRIP13 in human SCCHN. D’Silva and colleagues reported that TRIP13 was significantly up-regulated, as analyzed by TCGA and GEO data sets, in tumor tissues and was able to promote non-malignant cells malignant transformation, tumor growth and invasion. TRIP13 binds with KU70 and KU80 to activate DNA-PKc for DNA repairing, which help cells with DNA damage to escape cell cycle checkpoint and apoptosis. These cells tend to transformed into cancer cells [14]. In this study, we described a novel mechanism underlying TRIP13 promoting B-cell malignant transformation. TRIP13 knockdown induced an increasing expression of anti-apoptotic protein PUMA in p53-mutant Granta-519 and p53-wild JVM-2 cells. Generally speaking, the expression of PUMA is regulated by the tumor suppressor p53, and PUMA has been shown to be involved in p53-mediated apoptosis [23, 24]. However in CLL cells, PUMA tend to induce cell apoptosis through a TRIP13 dependent pathway. TRIP13 is a transcription factor that interacts with the ligand binding domain of the thyroid receptor (TR) as well as a variety of target genes. Unlike most TRIP proteins which function only in the presence of hormones, TRIP13 does not require the presence of thyroid hormone to interact with TR [25]. Hence, it is reasonable to hypothesize that TRIP13 interacts with PUMA promoter region to reduce PUMA transactivity following DNA damage and then protects B-cell from apoptosis. Chromatin immunoprecipitation (ChIP) and dual luciferase reporter assay can be performed to confirm the transcriptional regulatory relation between TRIP13 and PUMA.

MYC oncogene family members are broadly implicated in human cancers [26]. Over-expression of MYC promotes tumorigenesis by activating the transcription of target genes that drive cell proliferation and growth [27]. Among B-cell tumors, MYC has been historically implicated in the molecular pathogenesis of aggressive non-Hodgkin lymphomas [28]. However, there is increasing evidence that MYC has a critical role also in CLL, including the observations that: (i) a regulatory region centromeric to MYC contains multiple single nucleotide polymorphisms (SNPs) that have been implicated by genome wide association studies (GWAS) in susceptibility to CLL [29]; (ii) MYC is activated by the engagement of a number of CLL surface receptors that are known to be relevant for the leukemic clone, including the B-cell receptor, NOTCH and BAFF [21, 30, 31]; (iii) expression of MYC and its target genes is increased in high risk CLL and in tumor compartments containing proliferating CLL cells (i.e. the lymph node), and correlates with poor outcome [21, 32]; and (iv) transgenic mice expressing MYC together with BAFF develop a CLL-like disease [21]. In acute lymphocytic leukemia, decreased expression leads to an up-regulation of PUMA [33]. Moreover, loss of PUMA enhances c-MYC-driven B-cell lymphoma development in mice [34]. These evidences implied that c-MYC/PUMA axis is pivotal in CLL. In this study, we found that c-MYC promoted TRIP13 transactivity by binding to its promoter region directly. Grandori and colleagues reported that TRIP13 is a c-MYC dependent synthetic lethal gene in c-MYC over-expressed cancer cells [26]. Therefore, we hypothesized that c-MYC/TRIP13/PUMA play a critical role in promoting B-cell malignant transformation by inhibiting cell apoptosis.

CLL displays a heterogeneous clinical course, some patients living for years with asymptomatic disease and others experiencing early progression requiring therapeutic intervention. Hence, it is important to identify novel biomarker for CLL patients. Several researches focused on the genomic level [35]. However, the expression level of driver gene in CLL is also pivotal. The qPCR data of TRIP13 expression level in cell lines showed that not only CLL lines but also B-cell lymphoma cell lines had high TRIP13 mRNA level, which indicated that TRIP13 may be a broad-spectrum oncogene in B-cell lymphoma. Hence, TRIP13 expression detection in a wide range of B-cell lymphoma including Diffuse Large B Cell Lymphoma, Mantle Cell Lymphoma and Mucosa-associated Lymphoid Tissue Lymphoma is also meaningful. Since the research on TRIP13 is still little, there is no TRIP13 inhibitor under clinical development in our knowledge. Identifying the function of the TRIP13 protein in CLL cells is a significant advance. Having an existing chemical that blocks the protein could speed the process of moving to clinical trials.

DSB is the most dangerous type of DNA damage and is often induced by radiation and chemotherapy. Error-prone or excessive repair promotes mutations, chromosome instability and cancer, whereas unrepaired DNA leads to cell death [36]. In cancer, efficient repair of radiation- and chemotherapy-induced DSB promotes treatment resistance with subsequent relapse through non-homologous end joining (NHEJ) [37]. Therefore, further study is needed for the function of TRIP13 in chemo-resistance and the corresponding molecular mechanism in CLL.

In summary, our results suggest that TRIP13 plays an important role in CLL. Full understanding of the precise role of CLL in CLL may provide the opportunity to develop a novel therapeutic strategy by suppressing expression of CLL in CLL cells. In addition, TRIP13 has potential as a relevant clinical indicator of disease progression and as a prognostic marker for patient survival in CLL. Translational research on the clinical use of TRIP13 is required to generate a methodology and evaluate the molecular diagnostic ability of TRIP13 in CLL.

## MATERIALS AND METHODS

### CLL patient samples

CD19+ B cells was harvested and isolated from peripheral blood cells of 36 CLL patients and 33 healthy donors who were hospitalized in the affiliated Cancer Hospital of Zhengzhou University (Henan Cancer Hospital, Henan, China) between Mar 2014 and Oct 2015. CD19+ B cells from healthy donors and patients were isolated by magnetic cell sorting (MACS) of Lymphoprep-separated buffy coats using MACS CD19 Microbeads (Miltenyi Biotec, Bergisch Gladbach, Germany). Written informed consent was obtained from all patients. The Ethics Committee of the Henan Cancer Hospital approved the study protocol, and all experiments were performed in accordance with the approved guidelines.

### Cell lines

B-cell Lymphocytic Leukemia cells Granta-519, JVM-2 (American Type Culture Collection, USA) were cultured in DMEM (Gibco, USA) supplemented with 10% fetal calf serum (Thermo Fisher Scientific, USA) at 37 °C with 5% CO2.

### Real-time quantitative PCR (RT-qPCR)

RNA extraction was performed using TRIzol (Invitrogen, USA) according to the manufacturer's instructions. cDNA was synthetized by reverse transcription using an ABI 2720 thermal cycler (ABI Biosystems, USA) according to the manufacturer's instructions (M-MLV-RTase, Promega, USA). The cDNA product was detected using a SYBR Green Supermix kit (Toyobo, Osaka, Japan) with a Takara Bio PCR Thermal Cycler Dice Real Time TP800 (Takara, Japan). The cycling parameters were 95 °C for a 30-s hot start followed by 45 cycles of 95 °C for 5 s and 60 °C for 30 s. The relative mRNA expression in patients (Candidate gene/GAPDH) was determined using the 2^−ΔCt^ method. The relative mRNA fold change was determined using the 2^−ΔΔCt^ method.

### Western blot analysis

Whole-cell lysates were prepared with ice-cold radioimmunoprecipitation assay buffer (Sigma-Aldrich), quantified, and loaded onto SDS-PAGE. After electrophoresis, proteins in the gel were transferred to a nitrocellulose membrane and incubated with primary antibodies at 4°C overnight. Western blot was conducted using antibodies specific for TRIP13 (Abcam, (ab204331)), PUMA and GAPDH (glyceraldehyde 3-phosphate dehydrogenase) (CST, #12450; Santa Cruz Biotechnology, sc-25778), followed with horseradish peroxidase-labeled secondary antibody (Santa Cruz Biotechnology, sc-2004/sc-2005). Signals were detected by chemiluminescence (ECL Western Blotting Detection Reagents, GE Healthcare) and visualized using G:BOX Chemi Gel Documentation System (Syngene, Frederick, MD, USA).

### Plasmids and lentiviral transduction

The lentiviral vectors were purchased from Shanghai Genechem Company Ltd., China. shRNA sequence (5′-CCGG-GC target sequence-CTCGAG- reverse complementary target sequence GC-TTTTTG-3′) was subcloned into silencing vector. A scramble siRNA (5′-GCC TAA CTG TGT CAG AAG GAA-3′) was used as the negative control (NC). The siRNA targeting sequences of TRIP13 gene were 5′- TAC TCA ACA GAC ATA ATA T -3′. The siRNA targeting sequences of c-MYC gene were 5′-GCG AGG ATA TCT GGA AGA AAT-3′. The siRNA targeting sequences of PUMA gene were 5′-GGA GGG TCC TGT ACA ATC TCA-3′. C-MYC overexpression plasmid was constructed by subcloning c-MYC cDNA into GV365 plasmid (Shanghai Genechem Company Ltd., China). For TRIP13 promoter reporter plasmid, a 200bp containing two c-MYC binding sites (chr5: 884008-884028 strand (+) and chr5: 884117-884131 strand (-)) sequences were inserted into PGL3-promoter plasmid before SV40 promoter. The cells were infected with a MOI of 50∼100. Cells were incubated at 37 °C with 5% CO2 until they reached ∼40% confluence before infection.

### MTT assay of cell growth inhibition

Infected and noninfected CLL cells were seeded in 96-well plates at a density of 2 × 10^3^ cells/well and incubated at 37°C for 1, 2, 3, 4, and 5 days, respectively. Then, cells were washed two times with PBS and 3-(4,5-dimethyl-2-yl)-2,5-diphenyltetrazolium bromide (MTT) solution (5 mg/mL) was added to each well. After 4 hours of incubation, supernatants in each well were removed and then 100 μL of dimethyl sulfoxide (DMSO) was added to solubilize the formazan salt. Ten minutes later, the optical density (OD) was measured at 490 nm by using a microplate reader.

### Apoptosis assays

Apoptosis was assessed using annexin V-based flow cytometry using standard laboratory methodology. Briefly, cells were transfected as described above, and incubated for 5 days. The cells were then harvested, resuspended in binding buffer at a density of 1 × 10^6^ cells/ml, and 100 μl of this suspension was added to FACS tubes and stained with annexin V-APC. Cells were mixed gently in a dark room for 15 min at room temperature, and then analysed using flow cytometry. Although standard flow cytometry apoptosis assays use co-staining with propidium iodide, we analysed only annexin V since the cells were transfected with a EGFP-expressing vector and the excitation wavelengths of propidium iodide and EGFP overlap.

### BrdU assays

To measure proliferation, DNA synthesis was assessed by BrdU incorporation assay using a BrdU kit (Roche, USA) following the manufacturer's instructions, with the cells pulsed with BrdU for 12h at each time point.

### Caspase 3/7 activity assay

CLL cell Caspase 3/7 activity after TRIP13-KD and NC group were performed following the manual of Promega Caspase-Glo® 3/7 assay kit (Promega, USA). Briefly, TRIP13-KD and NC lentiverus infected cells were planted in 96 well plates for 3 days and 1 × 10^4^ cells were isolated and added to 100 μl Caspase-Glo buffer. After 300-500 rpm for 30 min, the samples were incubated in room temperature for 0.5 hours and assayed by a microplate reader.

### Microarray gene expression analysis

After infection with TRIP13-KD total RNA was extracted from JVM-2 cells, and 50–500 ng of RNA was used to generate biotin-modified amplified RNA (aRNA) using a GeneChip 3′ IVT Express Kit (Affymetrix, USA). Reverse transcription was performed using a T7 oligo (dT) primer and a first-strand IVT Labelling Master Mix was used to produce multiple copies of biotin-modified aRNA. The aRNA was then purified and quantified. After fragmentation the aRNA was hybridized to the GeneChip Human Genome U133 plus 2.0 Array (Affymetrix, USA). After hybridization the chips were stained with phycoerythrin and washed in a Genechip Fluidics Station 450. The microarray signals were scanned and analysed using a Genechip Array Scanner 3000 7G.

### Luciferase reporter assay

Cells cultured in 24-well plates were transfected with promoter reporter construct or mutant variants and overexpression or shRNA vector. A Renilla luciferase–containing plasmid, which is driven by thymidine kinase promoter, was always included in transfection to control transfection efficiency. Luciferase activity was determined by using a dual luciferase reporter assay system following the manufacturer's instructions (Promega, Madison, WI).

### Bioinformatics

Differentially expressed genes (DEGs) between the TRIP13-KD and NC groups with corrected p-values of < 0.05 and an absolute fold change of > 1.5 were considered to be significantly differentially expressed. Gene Ontology (GO) enrichment analysis and Kyoto Encyclopaedia of Genes and Genomes and BioCarta pathway databases were performed on significant DEGs. The microarray data were also analyzed by Ingenuity Pathway Analysis (IPA) and exported the “Canonical Pathway”, “Disease and Function”, “Upstream Analysis” and “Regulator Effects” reports.

### Statistical analysis

Statistical analyses were performed using GraphPad Prism and GraphPad InStat software (GraphPad Software, La Jolla, CA, USA). T-tests were used to determine significance, with a p-value of < 0.05 to indicate a significant difference. Data are presented as means ± SD (n=3).

## SUPPLEMENTARY MATERIALS FIGURES



## Abbreviations

TRIP13: Thyroid Hormone Receptor Interactor 13
CLL: Chronic Lymphocytic Leukemia
IPA: Ingenuity Pathway Analysis
SCCHN: Squamous Cell Carcinoma Of The Head And Neck
NC: Negative Control
KD: Knockdown
TR: thyroid receptor
ChIP: Chromatin immunoprecipitation
DEGs: Differentially expressed genes
GO: Gene Ontology
DMSO: Dimethyl Sulfoxide
OD: Optical Density
