# Supplementary material for: Loss of thyroid hormone receptor interactor 13 inhibits cell proliferation and survival in human chronic lymphocytic leukemia
Source: Oncotarget. 2017 Mar 9;8(15):25469–81. doi: 10.18632/oncotarget.16038 (PMC5421944; doi:10.18632/oncotarget.16038)
Supplement: Supplementary file 1 [file oncotarget-08-25469-s001.pdf]

Loss of thyroid hormone receptor interactor 13 inhibits cell proliferation and survival in human chronic lymphocytic leukemia

SUPPLEMENTARY MATERIALS

SUPPLEMENTARY FIGURES

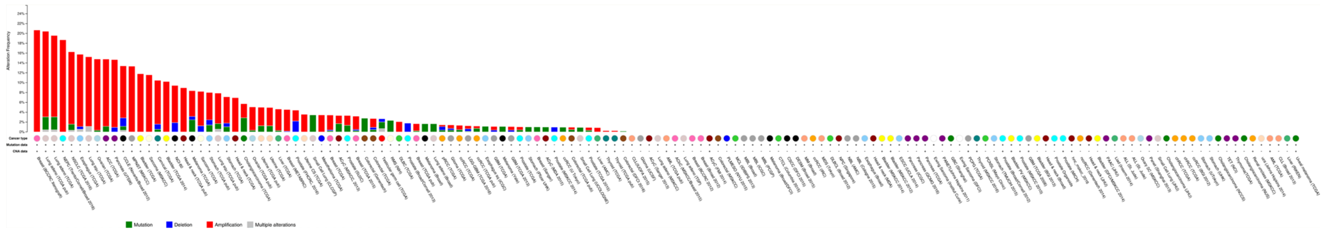

Supplementary Figure 1: Meta-analysis of TRIP13 mutation, DNA copy number and gene expression in multiple cancers using TCGA (http://www.cbioportal.org/).

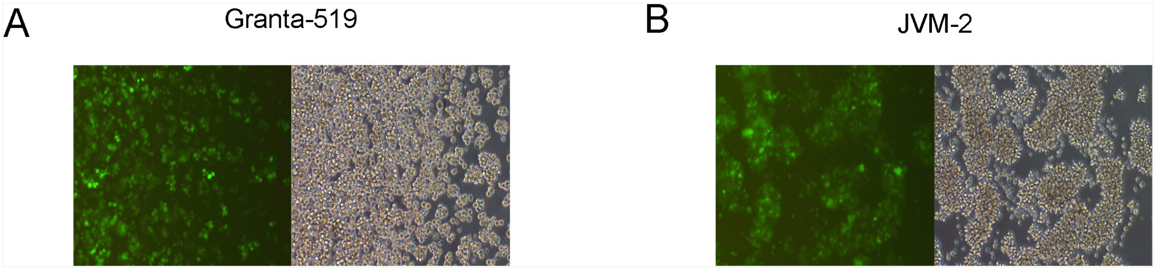

Supplementary Figure 2: Bright and fluorescence field of Granta-519 and JVM-2 cells after infection with lentivirus containing TRIP13 at magnification of ×100.

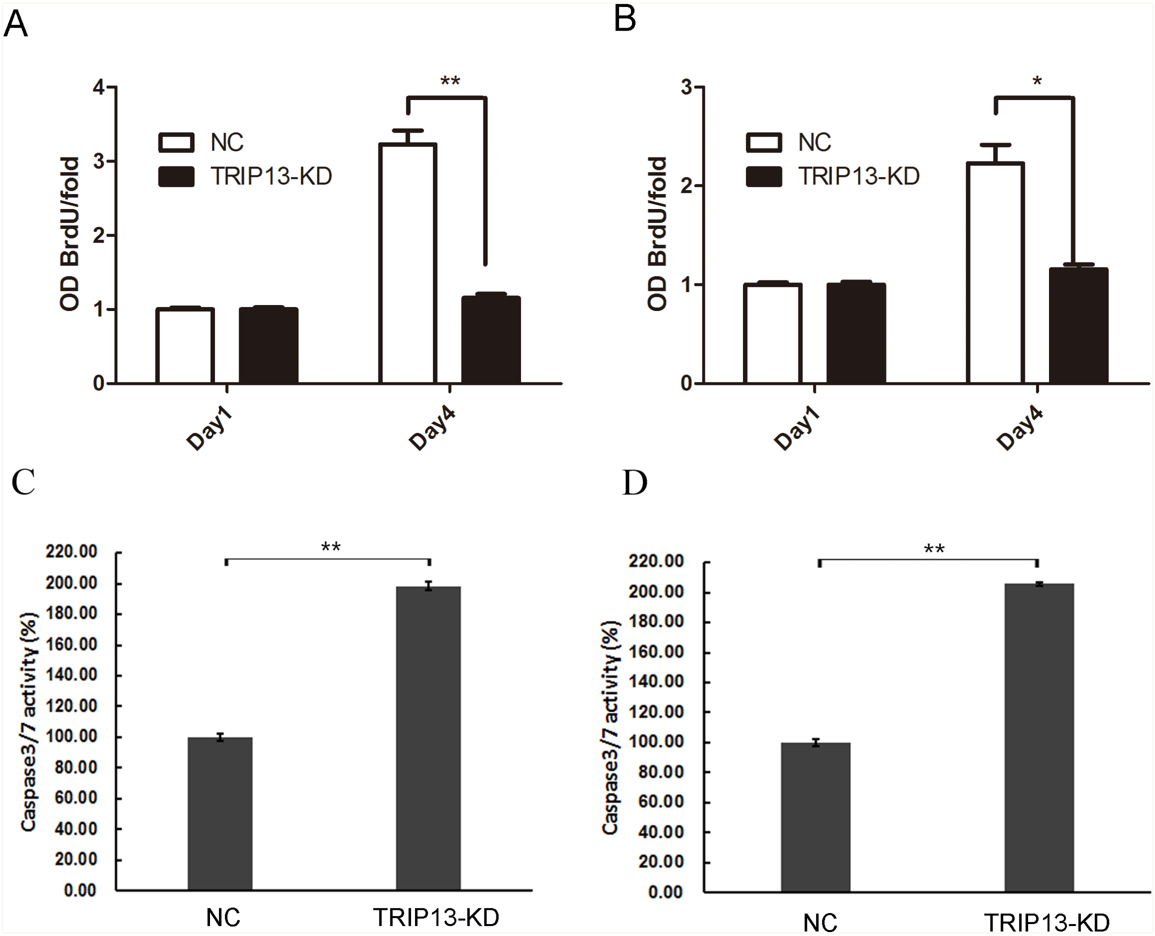

Supplementary Figure 3: BrdU assay showed the cell proliferation state in (A) Granta-519 and (B) JVM-2 cells after infection with lentivirus containing TRIP13 were detected. (*p<0.05, **p<0.01, ttest). Caspase 3/7 activities in **(C)** Granta-519 and **(D)** JVM-2 cells after infection with lentivirus containing TRIP13 were detected. (**p < 0.01, ttest).

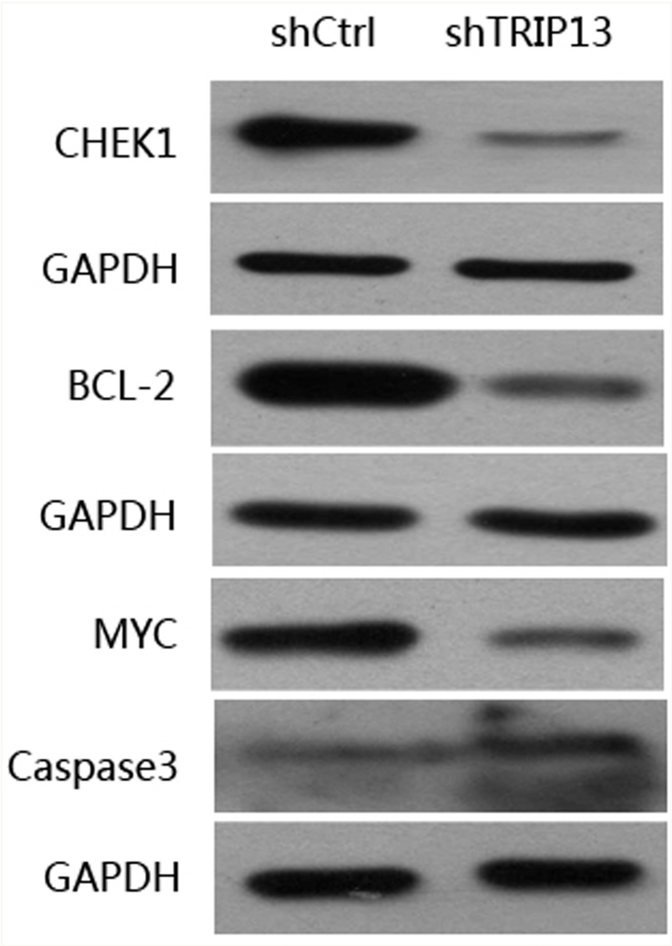

Supplementary Figure 4: Protein level of CHEK1, BCl-2, c-MYC and Caspase3 in NC and TRIP13 knockdown JVM-2 cells detected by western bolt.

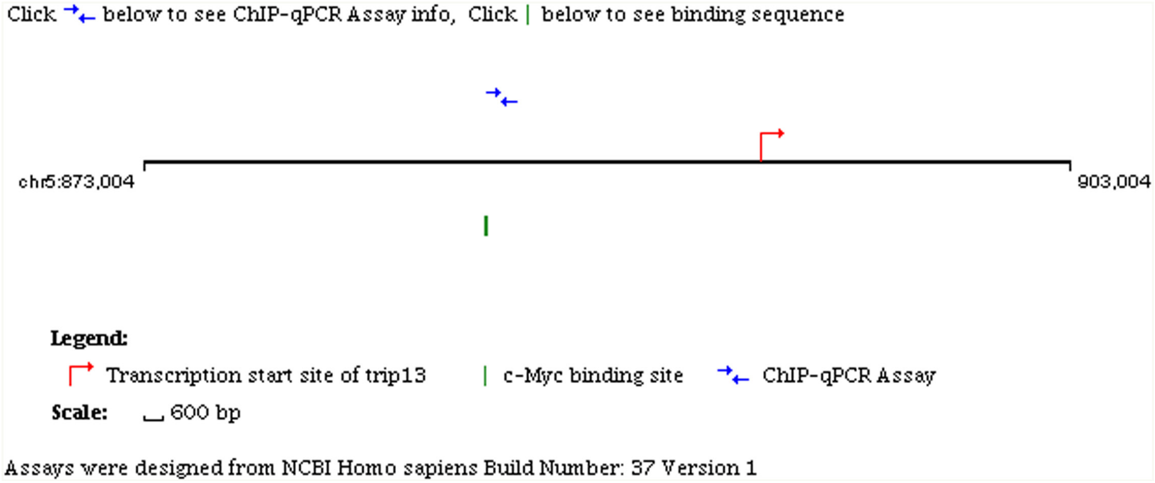

Supplementary Figure 5: Prediction of c-MYC binding sites upstream TRIP13 5UTR using The Champion ChiP Transcription Factor Search Portal.
